# Supplementary material for: StrainXpress: strain aware metagenome assembly from short reads
Source: Nucleic Acids Res. 2022 Jul 1;50(17):e101. doi: 10.1093/nar/gkac543 (PMC9508831; doi:10.1093/nar/gkac543)
Supplement: gkac543_Supplemental_Files [file gkac543_supplemental_files.zip › StrainXpress strain aware metagenome assembly from short reads supplementary.pdf]

StrainXpress: strain aware metagenome assembly from short reads Supplementary Material

Xiongbin Kang[1,†], Xiao Luo[1,†], Alexander Schönhuth[1,*]

[1] Genome Data Science, Faculty of Technology, Bielefeld University, Bielefeld, Germany

[†]These authors contributed equally to the work.
[*]To whom correspondence should be addressed.

SUPPLEMENT: DETAILS ON USING DE BRUIJN GRAPHS AND OVERLAP GRAPHS FOR STRAIN AWARE ASSEMBLY

For understanding some more helpful details, we recall the definition of a de Bruijn graph (DBG), where nodes reflect k-mers, that is substrings of reads of length k, and edges reflect overlaps of k-mers of exactly length k-1. Assemblers that make use of DBGs for the purposes of assembling reads (in contrast to just counting k-mers for the purposes of error correction, for example, which does not yet mean to follow the DBG paradigm), are supposed to follow the DBG assembly paradigm. Note that assembly tools that make use of k-mers for the purposes of error correction, without working with DBGs, do not necessarily follow the DBG assembly paradigm.

The second major assembly paradigm is referred to as "overlap-layout-consensus" (OLC) paradigm. Unlike for DBG assemblers, the central data structure that OLC assemblers make use of are overlap graphs. In overlap graphs, vertices r correspond to (full-length) reads, whereas (directed) edges (r_1,r_2) indicate that there is "substantial" overlap between the suffix of r_1 and the prefix of r_2. The exact quantification of "substantial" depends on the particular application and the characteristics of the sequencing technology in use.

To understand the issues that DBG assemblers are confronted with, one needs to realize that mutations that are genetically linked—hence appear in combination in identical strains—show as pairs, or even triples or quadruples and so on (when more than 2 mutations are linked) that co-occur across reads repeatedly. Because they co-occur, they are not as easily confounded with sequencing errors, which tend to show in isolation: repeatedly showing patterns of errors would contradict the assumption of independence of sequencing errors. This, however, is very uncommon for short reads.

In summary, one can tell true mutations from their patterns of co-occurrence, because patterns that recur across different reads contradict the assumption of independence, hence contradict that the mutations reflect sequencing errors.

Just as any other genome, strain-specific genomes are characterized by the patterns of mutations that they harbor. Therefore, the identification of patterns of co-occurring mutations that are characteristic of strains is a decisive task. Genomes of strains from identical species differ only by relatively small amounts of variations, such that the characteristic mutations can be spaced out by comparatively large amounts. Because the genetic linkage of mutations characteristic for strains can stretch across such large genomic distances, evaluating reads at their full length for such patterns is a major asset in strain aware assembly.

Understanding these genetic conditions now immediately elucidates why DBG assemblers can have decisive disadvantages over assemblers that leave sequencing reads intact. In short, DBG assemblers that operate with k-mer based DBGs isolate mutations that co-occur at a distance larger than k, hence break up the patterns of co-occurrence. This means a considerable drawback in comparison with tools that preserve information about co-occurrence of mutations across longer ranges of genomic distance, such as overlap graph based assemblers.

As explained above, isolating true mutations leads more easily to confounding them with sequencing errors when evaluating mutations on a solid statistical basis. The consequences of this confusion is to remove them upon their identification. In some more detail, note that in DBGs, mutations that are either genuinely isolated, or that co-occur at a distance larger than k, show as isolated "bubbles". It is common to just remove these "bubbles" for the purposes of correcting errors (1). As a consequence, several genomes may be collapsed into one, because the bubbles that indicated their crucial differences had been removed. This procedure potentially annihilates the identity of strains. In an extreme case, genomes are not just collapsed, but disappear entirely, because all of the characteristic mutations had been taken for errors, which may happen for low-frequency strains in particular. Note that, however, very often low-frequency strains are the ones of greatest interest in clinical settings, because inducing resistance to treatment, or being particularly pathogenic.

For OLC based, or just overlap graph based assemblers this class of issues is considerably less expressed, because they preserve information on genetic linkage of strain-specific mutations to the largest degree possible. Therefore, we recall that unlike DBGs, overlap graphs capture sequencing reads at their full length, which justifies the value of these graphs with respect to the issues just raised. Despite this natural advantage, however, establishing and processing overlap graphs lacks the elegant solutions that DBGs can rely on. Nevertheless, because of the clear limitations of DBG based assembly, the benefits of overlap graph based assembly in strain aware assembly are strikingly obvious, which justifies to pursue related approaches further (as we do here).

USING XC AS PREPROCESSING TOOL: EVALUATION OF ASSEMBLIES AND RUNTIME.

We evaluated the quality of the assemblies resulting from using XC as a preprocessing tool in direct comparison with the original approaches, see Table 1 in the main text for the corresponding evaluation. Note that OGRE (the original counterpart of XC) was suggested to be used as preprocessing tool for (potentially strain aware) metagenome assemblers. So it made sense to analyze XC as a general pre-processing.

For both MEGAHIT and IDBA-UD preprocessing data using XC improves the quality of the resulting assemblies. Further, while assembly performance of GATB-Minia was not influenced by using XC, the assemblies of SPAdes get rather worse by pre-processing the input with XC. Note that reasonable explanations are difficult to provide, because the devil is in the details for each of the competing methods.

In addition, we also measured the CPU time and peak memory usage for the different approaches before and after combining XC on a x86_64 GNU/Linux machine with 48 CPUs. The results are reported in Supplementary Table S4. Evidently, MEGAHIT is the fastest tool due to it can efficiently parallel construct a succinct de Bruijn Graphs to assemble reads. Beside XC + SPAdes, other methods combined with XC spent more CPU time, but the peak memory is less than before. It means that construct one de Bruijn Graphs for all reads is more time efficient rather than build them in small clusters separately. However, many clusters with low depth cannot complete assembly by SPAdes.

ALTERNATIVE METHODS: UNRESOLVED ERRORS

StrainFinder: (KeyError: 'lacto_dnaG') was thrown. Like other researchers before who encountered the exact same problem (the issue so far unresolved https://github.com/cssmillie/StrainFinder/issues/3).

ConStrains: (Error: Unable to find the mpa_pkl file at: mpa_v20_m200.pkl.) when trying to run it. We believe that the issue is rooted in using MetaPhlAn2, which establishes a dependency on third-party software. Just as we did, other researchers / applicants encountered the same problem. See (https://forum.biobakery.org/t/unable-to-find-the-mpa-pkl/503/2) for documentation of the issue.

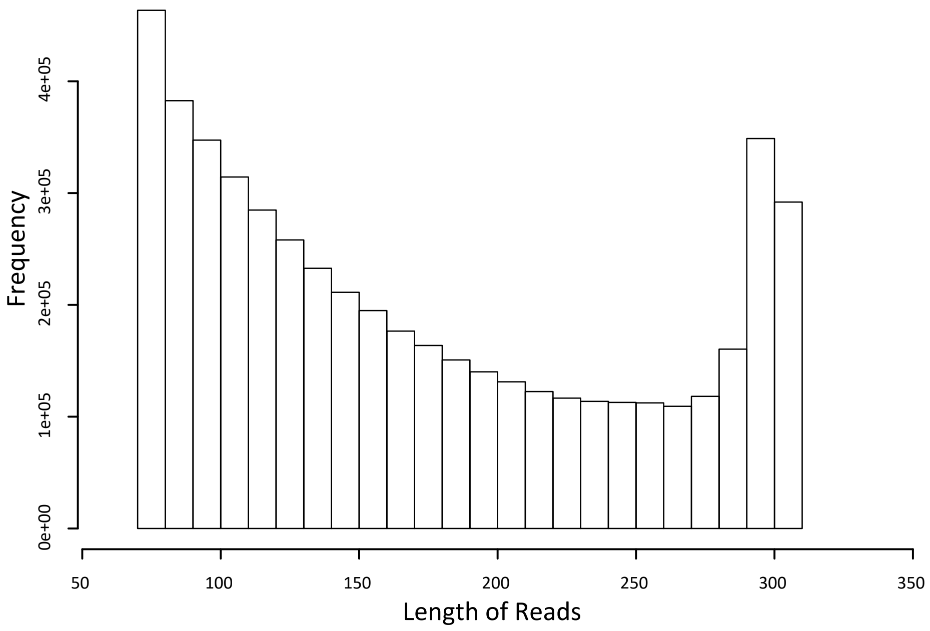

Figure S1. The reads length of NWCs data set

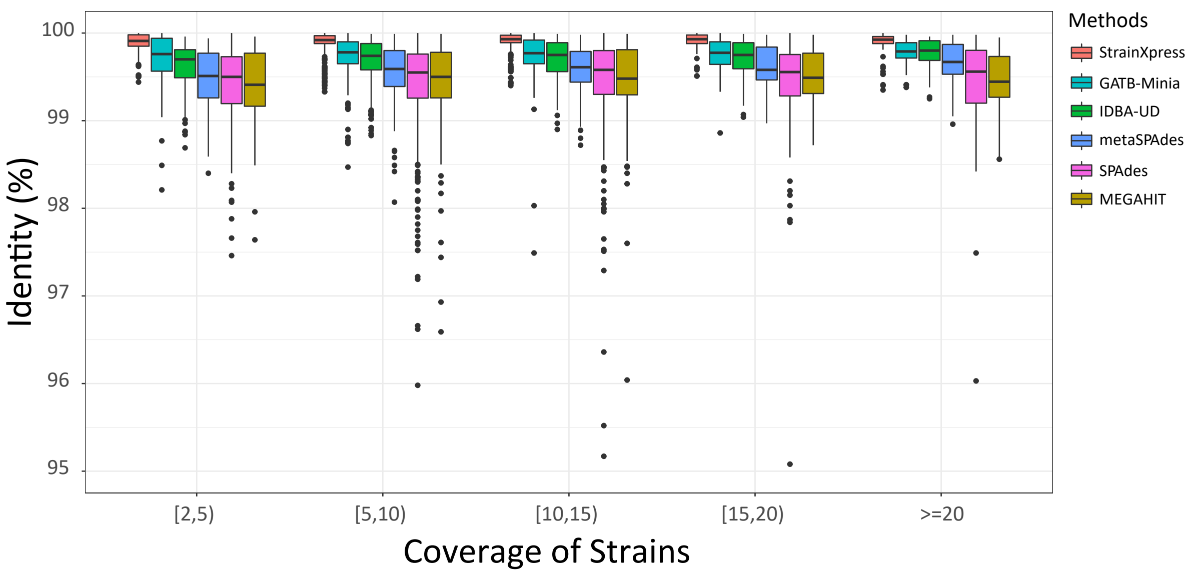

Figure S2. Identity between contigs and real genomes. Contigs were uniquely mapped the reference and then calculate the percentage of completely match base in the total base of the contigs. Here we estimated the identity for the 1057 strains in the high complexity set (2X250bp), and then analyzed the influence of strain coverage.

Table S1. The GenBank number of reference genomes. Samples information and assembly results of real gut metagenome sequencing data.

| Supplementary Table S1.xlsx |

Table S2. Comparing running times of clustering and local assembly in StrainXpress with OGRE and POLYTE on the low complexity data set (reads length 2X250 bp). XC is short for the clustering step of StrainXpress, while XLA is short for the local assembly step of StrainXpress.

Methods	CPU Time (h)	Cluster Number
OGRE	84.07	568
XC	1.82	899
POLYTE	188.92	—
XLA	22.55	—

Table S3. Comparison of performance rates when replacing the clustering step of StrainXpress (XC) and the local assembly step of StrainXpress (XLA) with OGRE and POLYTE, respectively. In all cases, global assembly follows to generate output.

Assembly	XC + XLA	OGRE + XLA	XC + POLYTE	OGRE + POLYTE
N50	2754	2759	2461	2432
Misassembled Contigs (%)	0.11	0.061	0.068	0.08
Genome Fraction (%)	93.45	93.30	94.50	94.38
N Rate	0	0	0	0
Error Rate	0.06	0.06	0.05	0.05

Table S4. Running times of StrainXpress, original alternative approaches, and alternative approaches when preprocessing data using the clustering routine implemented in StrainXpress (XC) on the medium complexity data set (reads length 2X250 bp). In these methods combinating with XC, the peak memory usage represents the peak memory when they assemble reads in small clusters.

Asembly	CPU Time (h)	Peak Memory Usage (GB)
StrainXpress	228.19	4.60
SPAdes	210.07	42.13
XC + SPAdes	122.63	0.68
GATB-Minia	299.48	15.11
XC + GATB-Minia	4972.95	5.48
IDBA-UD	96.16	36.75
XC + IDBA-UD	211.42	0.61
MEGAHIT	**28.57**	6.17
XC + MEGAHIT	223.90	**0.28**

Table S5. Coverage and average nucleotide identity (ANI) of strains in the NWc data set.

Genomes	GenBank no	Coverage	ANI (%)
Streptococcus_thermophilus_isolate_NWC_1_1	CP029252.1	56.29	99.99
Streptococcus_thermophilus_isolate_NWC_2_1	CP031021.1	55.07	
Lactobacillus_delbrueckii_subsp.lactis_isolate_NWC_1_2	CP029250.1	39.38	99.24
Lactobacillus_delbrueckii_subsp.lactis_isolate_NWC_2_2	CP031023.1	35.13	
Lactobacillus_helveticus_isolate_NWC_2_4	CP031018.1	17.59	98.03
Lactobacillus_helveticus_isolate_NWC_2_3	CP031016.1	10.27	

Table S6. The Genome Fraction for the individual strains of the NWC data set. Length of reads >70 bp

GenBank Number of Strains<br>Coverage	CP029252.1<br>56.29X	CP031021.1<br>55.07X	CP029250.1<br>39.38X	CP031023.1<br>35.13X	CP031018.1<br>17.59X	CP031016.1<br>10.27X	Average<br>35.62X
StrainXpress	**92.66**	**91.58**	**81.25**	**69.86**	65.22	**37.63**	**73.03**
MEGAHIT	89.87	84.04	57.82	42.98	**69.08**	31.47	62.54
IDBA-UD	87.52	82.30	70.98	27.12	68.79	23.94	60.11
SPAdes	86.12	80.66	79.00	20.41	66.83	25.67	59.78
GATB-Minia	83.48	78.12	74.73	29.87	66.83	22.29	59.22

Table S7. The Genome Graction of different strains in NWCs dataset. The length of reads >150 bp .

GenBank Strain Identifier<br>Coverage	CP029252.1<br>38.36X	CP031021.1<br>37.42X	CP029250.1<br>29.88X	CP031023.1<br>26.6X	CP031018.1<br>13.07X	CP031016.1<br>7.46X	Average<br>25.47X
StrainXpress	**97.28**	**94.57**	**84.15**	**72.49**	**68.71**	**39.59**	**76.13**
MEGAHIT	90.81	79.59	66.19	36.54	65.08	32.07	61.71
IDBA-UD	84.01	79.14	70.13	24.2	63.92	26.11	57.92
SPAdes	86.53	76.47	79.24	21.28	68.28	24.89	59.45
GATB-Minia	87.8	76.4	74.34	26.6	60.66	24.16	58.33

Table S8. Processing a simulated data that contains three high identity Salmonella strains to compare the assembly results of StrainXpress and Gretel. In here, SPAdes+Gretel denotes that the reference of Gretel is the contigs that were generated by SPAdes. MEGAHIT+Gretel denotes that the reference of Gretel is the contigs that were generated by MEGAHIT.

Methods (3 Salmonella)	StrainXpress	SPAdes	SPAdes+Gretel	MEGAHIT	MEGAHIT+Gretel
Genome Fraction (%)	90.84	61.21	83.78	66.09	**92.54**
Duplication Rate	1.21	1.04	**8.16**	1.02	**9.66**
Total Assembly Length	15044167	8206084	91869461	9315296	123285252
N50	2329	1664	2553	7647	6911
NGA50	2645	688	6977	3603	19966
Misassembled Contigs (%)	0.06	0.08	0.46	0.68	0.68
Identity (%)	**99.93**	99.85	97.70	99.61	97.12
Error Rate (%)	**0.059**	0.148	1.164	0.289	1.861
CPU Time (h)	2.24	1.23	**108.08**	0.95	**103.56**
Peak Memory Usage (GB)	1.26	2.88	1.50	**0.31**	11.27

Table S9. Identity between contigs and real genomes. We uniquely map the assembly results to the reference and then calculate the percentage of completely match base in the total base of the contigs.

Strains	StrainXpress	GATB-Minia	SPAdes	metaSPAdes	IDBA-UD	MEGAHIT
Bmock12 (Identity between contigs and real genomes %)						
Cohaesibacter sp. ES.047	99.96	100.00	100.00	99.99	100.00	99.97
Halomonas sp.HL-4	99.94	99.95	99.94	99.69	99.89	99.74
Halomonas sp.HL-93	99.93	99.97	99.95	99.88	99.92	99.77
Marinobacter sp.LV10MA510-1	99.92	99.95	99.98	99.98	100.00	99.85
Marinobacter sp.LV10R510-8	99.97	99.99	100.00	99.97	100.00	99.96
Micromonospora echinaurantiaca	99.98	99.75	99.99	99.98	99.99	99.78
Micromonospora echinofusca	99.83	99.79	99.98	99.97	99.99	99.72
Muricauda sp.ES.050	99.95	100.00	100.00	99.99	100.00	99.84
Propionibacteriaceae bacterium	99.99	100.00	100.00	100.00	100.00	99.95
Psychrobacter sp.LV10R520-6	99.84	100.00	100.00	99.96	100.00	99.96
Thioclava sp.ES.032	100.00	100.00	100.00	100.00	100.00	100.00
NWCs (Identity between contigs and real genomes %)						
Lactobacillus_delbrueckii_NWC_1_2	99.63	98.84	99.68	99.97	98.91	97.39
Lactobacillus_delbrueckii_NWC_2_2	99.69	99.14	99.70	99.61	97.82	96.97
Lactobacillus_helveticus_NWC_2_3	99.52	98.58	98.17	99.21	97.69	97.26
Lactobacillus_helveticus_NWC_2_4	99.80	97.58	98.83	99.60	98.77	97.07
Streptococcus_thermophilus_NWC_1_1	99.18	99.39	99.88	99.94	99.94	99.16
Streptococcus_thermophilus_NWC_2_1	99.03	99.72	99.63	98.98	98.91	98.94

Table S10. The parameters of the 5 assemblers base on recommendations in their manual and default values.

<table>
<tr><td>Methods</td><td>Reads Length 2X250 bp</td><td>Reads Length 2X150 bp</td><td>Reads Length 2X100 bp</td></tr>
<tr><td>IDBA-UD</td><td colspan="3">fq2fa fq_file fa_file; idba_ud -r fa_file –pre_correction -o result</td></tr>
<tr><td>GATB-Minia</td><td colspan="3">gatb –12 fq_file</td></tr>
<tr><td>MEGAHIT</td><td colspan="2">megahit –k-list 21,29,39,59,79,99,119,141 –12 fq_file -o reulst</td><td>megahit –k-list 21,29,39,59,79,99 –12 fq_file -o reulst</td></tr>
<tr><td>SPAdes</td><td colspan="2">spades.py -k 21,33,55,77,99,127 –careful –12 fq_file -o result</td><td>spades.py -k 21,33,55,77,99 –careful –12 fq_file -o result</td></tr>
<tr><td>metaSPAdes</td><td colspan="2">spades.py –meta -k 21,33,55,77,99,127 –12 fq_file -o reulst</td><td>spades.py –meta -k 21,33,55,77,99 –12 fq_file -o reulst</td></tr>
</table>

REFERENCES

1. Miller, J. R., Koren, S., and Sutton, G. (2010) Assembly algorithms for next-generation sequencing data. *Genomics,* **95**(6), 315–327.
